# Fractal time series analysis of postural stability in elderly and control subjects

**DOI:** 10.1186/1743-0003-4-12

**Published:** 2007-05-01

**Authors:** Hassan Amoud, Mohamed Abadi, David J Hewson, Valérie Michel-Pellegrino, Michel Doussot, Jacques Duchêne

**Affiliations:** 1 Institut Charles Delaunay, FRE CNRS 2848, Université de technologie de Troyes, 10000 Troyes, France

## Abstract

**Background:**

The study of balance using stabilogram analysis is of particular interest in the study of falls. Although simple statistical parameters derived from the stabilogram have been shown to predict risk of falls, such measures offer little insight into the underlying control mechanisms responsible for degradation in balance. In contrast, fractal and non-linear time-series analysis of stabilograms, such as estimations of the Hurst exponent (H), may provide information related to the underlying motor control strategies governing postural stability. In order to be adapted for a home-based follow-up of balance, such methods need to be robust, regardless of the experimental protocol, while producing time-series that are as short as possible. The present study compares two methods of calculating H: Detrended Fluctuation Analysis (DFA) and Stabilogram Diffusion Analysis (SDA) for elderly and control subjects, as well as evaluating the effect of recording duration.

**Methods:**

Centre of pressure signals were obtained from 90 young adult subjects and 10 elderly subjects. Data were sampled at 100 Hz for 30 s, including stepping onto and off the force plate. Estimations of H were made using sliding windows of 10, 5, and 2.5 s durations, with windows slid forward in 1-s increments. Multivariate analysis of variance was used to test for the effect of time, age and estimation method on the Hurst exponent, while the intra-class correlation coefficient (ICC) was used as a measure of reliability.

**Results:**

Both SDA and DFA methods were able to identify differences in postural stability between control and elderly subjects for time series as short as 5 s, with ICC values as high as 0.75 for DFA.

**Conclusion:**

Both methods would be well-suited to non-invasive longitudinal assessment of balance. In addition, reliable estimations of H were obtained from time series as short as 5 s.

## Background

The study of balance deficits is of interest for many reasons, in particular for people with various pathological conditions affecting balance, and the elderly. In respect to an elderly population, falls are a major problem, in terms of both frequency and consequences. In France alone, more than two million falls are recorded among the elderly each year, leading to more than 9000 deaths [1]. Most prospective studies have attempted to identify risk factors, particularly in groups at high risk of falling [2-5]. The factors identified in these studies have often varied, mainly due to differences in methodology, diagnosis, and the study population [6]. Nevertheless, several factors are regularly cited, such as muscular weakness, a previous fall, or balance problems [2,4,7-9]. In addition, several factors that augment the risk of falling, such as visual, vestibular, or proprioceptive problems, will adversely affect balance [10-12].

Balance can be evaluated either clinically, using tests such as the "Timed Get-up-and-go" [13], "Berg Balance Scale" [14] and the "Tinetti Balance Scale" [15], or biomechanically, using a force plate to evaluate postural sway [16]. In order to measure postural sway, the movement of the centre of pressure (COP) over the support base of the subject can be evaluated [17], with the resulting stabilogram displaying the movement of the COP over time for anteroposterior (AP), mediolateral (ML), and resultant (R) directions. Simple statistical parameters derived from the stabilogram, such as the area and the shape covered by the displacement of the COP have been shown to predict risk of falls [3,18].

Although both clinical and biomechanical tests have been shown to be able to identify elderly at a greater risk of falling, such tests have yet to be used for long-term monitoring of balance. Recent technological advances might enable biomechanical tests to be used for home-based longitudinal study aimed at fall prevention. Before any such study could be envisaged there are several factors that need to be addressed. Firstly, the simple statistical parameters derived from the stabilogram offer little insight into the underlying control mechanism that is responsible for the degradation in balance observed. In addition, the duration of the testing remains problematic, with tests lasting longer than 10 s likely to decrease subject compliance. Finally, the testing equipment needs to be adapted for home-based non-invasive monitoring. The present study will address those issues related to the type of parameters that can be extracted from the stabilogram, as well as the shortest possible signal duration from which reliable parameters are able to be extracted. Information related to the development of a home-based assessment protocol can be found in [19].

In terms of the extraction of parameters that provide information related to underlying physiological control processes, over the last ten years, a number of authors have used more complex signal processing techniques to analyse the stabilogram (signal). These techniques have included Stabilogram Diffusion Analysis (SDA) [20-22], Detrended Fluctuation Analysis (DFA) [23,24], and Rescaled Range Analysis (R/S) [23]. Such methods have been used as the stabilogram has been shown to be a nonstationary time series [25,26] that displays fractal characteristics [21,27]. The advantage of such methods is that information related to the underlying motor control strategies governing postural stability could be extracted. For instance, the SDA, DFA, and R/S methods provide information on the long-term correlations contained within the time series. Despite the unpredictability of fractal signals, an element of order can exist. This order, although not evident for two successive values, implies that values depend on the global history of the series, and that long-term correlations exist. Furthermore, such long-term correlations exhibit scaling laws, first described by Mandelbrot and Van Ness [28] and termed fractional Brownian motion in the following equation [28]:

⟨Δ*x*^2^⟩ ∝ Δ*t*^2*H*^

where Δx is the distance between two points separated in time by Δt, and where the Hurst exponent H is in the range 0 < H < 1.

When consecutive values are positively correlated (H > 1/2), the signal is said to show persistence, whereas negative correlations (H < 1/2) are termed anti-persistence. The special case of Brownian motion occurs when H = 1/2. The determination of the scaling exponent H of a stabilogram is of particular interest, as it can be inferred to relate to mechanisms of postural control [29].

The control of posture is very complex, involving input from the visual, vestibular, and proprioceptive systems. Collins and De Luca [30] suggested that both closed-loop and open-loop mechanisms of postural control are present in order to control postural sway. A closed-loop system implies that the system responds quickly to feedback concerning deviations from acceptable limits, and responds accordingly. In contrast, an open-loop system operates without feedback, and is therefore much less accurate than a closed-loop system. Collins and De Luca identified two distinct zones in their stabilogram diffusion plots, each of which had a different scaling exponent [30]. They interpreted the presence of short-range positive correlations (H > 1/2) in COP data as verifying the use of open-loop control mechanisms over short time periods (t < 1 s). Thereafter, long-range negative correlations were observed (H < 1/2). The explanation proposed was that posture is loosely controlled until acceptable limits are passed, upon which time a more rigid closed-loop system is applied, ensuring that postural sway values fall within more acceptable limits. The point at which these two strategies converge, the "critical time" gives an indication of the degree of laxity in control. In a subsequent study, Collins and colleagues observed longer critical times in elderly subjects, implying that a greater time spent in open-loop control could be a factor in falls in the elderly [29].

Since the pioneering work of Collins and De Luca, subsequent studies, in particular that of Delignieres and colleagues [23], have failed to find any evidence of two distinct zones of control. They suggested that the results of Collins and colleagues were due to the manner in which the biological time series was mapped as a stochastic process, and the resulting estimations of H. The method of Collins and De Luca did not take into account that biological time series have bounds imposed by physiological limits, as compared with fractional Brownian motion, which is unbounded and can therefore be expected to increase indefinitely with time. However, the upper limit imposed on the COP displacement by the support area of the feet acts as a ceiling which causes the second anti-persistent part of the stabilogram diffusion plot [23]. When there is a definite upper limit for a time series, scaling is restricted to short time intervals, beyond which values saturate at twice the variance of the data [31]. The two methods previously cited to calculate the Hurst exponent, DFA and R/S use an integrated signal, and therefore do not suffer from the bounded limitation of the second part of SDA. However, the choice of methods depends of the nature of series to which the methods are to be applied. The DFA method can be applied to both fractional Brownian motion (fBm) and fractional Gaussian motion (fGn) whereas R/S can only be applied to fGn series [32]. It is necessary, therefore to apply the DFA method first, from which the nature of the time series can be determined. If the slope α obtained from DFA is greater than 1, this indicates that the series is fBm; if α is less than 1, the series is fGn. In the present study, α obtained from DFA was greater than 1 for all subjects, thus all time series are fBm and the R/S method can not be used.

The aims of the current investigation are twofold: firstly, the SDA and DFA methods of estimating the Hurst exponent will be compared and applied to postural signals for elderly and control subjects. Secondly, the minimum recording duration needed in order to obtain reliable results will be identified for both methods.

## Methods

### Subjects

Ninety young control subjects and ten elderly subjects participated in the study. Anthropometric data for the two subject groups are presented in Table 1. All subjects who participated gave their written informed consent. No subjects reported any musculoskeletal or neurological conditions that precluded their participation in the study.

**Table 1:** Anthropometric data for elderly and control groups.

	Gender	Number	Age	Height	Weight
Control	Men	57	19.8 ± 0.9	179.5 ± 8.2	71.6 ± 9.9
	Women	33	19.6 ± 0.8	166.5 ± 4.9	58.9 ± 8.1
Elderly	Men	4	80.0 ± 2.2	173 ± 4.5	81.9 ± 8.5
	Women	6	80.8 ± 6.0	160.5 ± 1.2	68.4 ± 5.8

Values are means and standard deviations.

### Centre of pressure data

Centre of pressure data were recorded using a Bertec 4060-08 force plate (Bertec Corporation, Columbus, OH, USA), which amplifies, filters, and digitises the raw signals from the strain gauge amplifiers inside the force plate. The resulting output is a six-channel 16-bit digital signal containing the forces and moments in the *x*, *y*, and *z *axes. The digital signals were subsequently converted via an external analogue amplifier (AM6501, Bertec Corporation). The initial COP signals were calculated with respect to the centre of the force-plate before normalization by subtraction of the mean.

### Data acquisition and processing

Data were recorded using the ProTags™ software package (Jean-Yves Hogrel, Institut de Myologie, Paris, France) developed in Labview^® ^(National Instruments Corporation, Austin TX, USA). Data were sampled at 100 Hz, with an 8^th^-order low-pass Butterworth filter with a cut-off frequency of 10 Hz. All calculations of COP data were performed with Matlab^® ^(Mathworks Inc, Natick, MA, USA).

### Experimental protocol

All subjects were tested either barefoot or wearing socks, and were instructed to stand upright with their arms by their sides in front of the force-plate, while looking at a target of a 10-cm cross fixed on the wall two meters in front of the force-plate. Upon a verbal command, subjects stepped onto the force plate, with no constraint given over foot position. Data were recorded for 30 seconds, which included both the step onto and off the force plate, and at least 20 seconds during which time subjects remained stationary in an upright posture. At the end of the trial another verbal command was given for subjects to step off the force-plate. Subjects performed the test four times, with a delay of 10 s between tests.

This protocol is similar to that which would be used for home monitoring, in that subjects were free to choose their foot position, the speed at which they stepped onto the force plate, and the length of their step onto the force plate.

### Estimation of the Hurst exponent

#### Stabilogram Diffusion Analysis (SDA)

Collins and De Luca [30] hypothesized that the trajectory of the COP could be modelled as a correlated random walk. They proposed a simple method to calculate the scaling exponent *H *of a stabilogram, whereby the square of the displacement for a given time interval Δt is calculated for all possible pairs of points separated by Δt, and the average calculated as:

〈Δx2〉Δt=1N−m∑(xi+Δti=1N−m−xi)2
 MathType@MTEF@5@5@+=feaafiart1ev1aaatCvAUfKttLearuWrP9MDH5MBPbIqV92AaeXatLxBI9gBaebbnrfifHhDYfgasaacH8akY=wiFfYdH8Gipec8Eeeu0xXdbba9frFj0=OqFfea0dXdd9vqai=hGuQ8kuc9pgc9s8qqaq=dirpe0xb9q8qiLsFr0=vr0=vr0dc8meaabaqaciaacaGaaeqabaqabeGadaaakeaadaaadaqaaiabfs5aejabdIha4naaCaaaleqabaGaeGOmaidaaaGccaGLPmIaayPkJaWaaSbaaSqaaiabfs5aejabdsha0bqabaGccqGH9aqpdaWcaaqaaiabigdaXaqaaiabd6eaojabgkHiTiabd2gaTbaadaWfWaqaamaaqaeabaGaeiikaGIaemiEaG3aaSbaaSqaaiabdMgaPjabgUcaRiabfs5aejabdsha0bqabaaabeqab0GaeyyeIuoaaSqaaiabdMgaPjabg2da9iabigdaXaqaaiabd6eaojabgkHiTiabd2gaTbaakiabgkHiTiabdIha4naaBaaaleaacqWGPbqAaeqaaOGaeiykaKYaaWbaaSqabeaacqaIYaGmaaaaaa@51BE@

where N is the number of points in the vector *x*, and *m *is the interval between two values expressed as the number of data.

Estimations of the Hurst exponent are then obtained from the graph of Δt by <Δx>^2 ^in log scale by calculating the slope of the short-term (H_S_) and long-term (H_L_) regions of the curve. The equations used by Collins and colleagues to estimate H_S _and H_L _contain several assumptions. The second derivative of the Δt by <Δx>^2 ^data is used to locate four times (T_1_, T_2_, T_3_, T_4_) between which the slopes H_S _(T_1_, T_2_) and H_L _(T_3_, T_4_) are calculated. The first time, T_1_, is always taken as zero, while T_2 _is the first maximum that occurs before 1 s. The slope H_S _is then calculated between these points. Similarly, the slope H_L _is calculated between T_3 _and T_4_, where T_3 _is calculated as the second maximum, and T_4 _as the first maximum occurring after the first minimum when the signal is analyzed backwards from 9 s. If no maximum is found before 7 s, T_4 _is taken as 9 s. A copy of the Matlab^® ^program, as well a detailed explanation are available at colleagues [33]. In order to estimate H_L _for the 5-s and 2.5-s windows, if the second maximum was not found, T_3 _was taken as 2.5 s for the 5-s window and 1.25 s for the 2.5-s window, while T_4 _was taken as 4.5 s and 2.5 s respectively, as these windows were too small to use the normal method of obtaining T_4 _between 7 and 9 s.

### Detrended Fluctuation Analysis (DFA)

Peng and colleagues [34] introduced another method of estimating the Hurst exponent specifically for biological time series data, which they termed Detrended Fluctuation Analysis (DFA). The first step is to subtract the mean from the original series, which is then integrated:

y(k)=∑i=1k[x(i)−x_]
 MathType@MTEF@5@5@+=feaafiart1ev1aaatCvAUfKttLearuWrP9MDH5MBPbIqV92AaeXatLxBI9gBaebbnrfifHhDYfgasaacH8akY=wiFfYdH8Gipec8Eeeu0xXdbba9frFj0=OqFfea0dXdd9vqai=hGuQ8kuc9pgc9s8qqaq=dirpe0xb9q8qiLsFr0=vr0=vr0dc8meaabaqaciaacaGaaeqabaqabeGadaaakeaacqWG5bqEcqGGOaakcqWGRbWAcqGGPaqkcqGH9aqpdaaeWbqaamaadmaabaGaemiEaGNaeiikaGIaemyAaKMaeiykaKIaeyOeI0YaaCbiaeaacqWG4baEaSqabeaacqGGFbWxaaaakiaawUfacaGLDbaaaSqaaiabdMgaPjabg2da9iabigdaXaqaaiabdUgaRbqdcqGHris5aaaa@43A7@

This series is then divided into windows of equal length *n*. If the total length *N *is not divisible by *n*, the length *N *is adjusted to the largest multiple of *n < N*. The local trend of each window y_n _is obtained and subtracted from the summed series, using a line of least-squared fit to obtain the detrended fluctuation *F(n) *as:

F(n)=1N∑k=1N[y(k)−yn]2
 MathType@MTEF@5@5@+=feaafiart1ev1aaatCvAUfKttLearuWrP9MDH5MBPbIqV92AaeXatLxBI9gBaebbnrfifHhDYfgasaacH8akY=wiFfYdH8Gipec8Eeeu0xXdbba9frFj0=OqFfea0dXdd9vqai=hGuQ8kuc9pgc9s8qqaq=dirpe0xb9q8qiLsFr0=vr0=vr0dc8meaabaqaciaacaGaaeqabaqabeGadaaakeaacqWGgbGrcqGGOaakcqWGUbGBcqGGPaqkcqGH9aqpdaGcaaqaamaalaaabaGaeGymaedabaGaemOta4eaamaaqadabaGaei4waSLaemyEaKNaeiikaGIaem4AaSMaeiykaKIaeyOeI0IaemyEaK3aaSbaaSqaaiabd6gaUbqabaGccqGGDbqxdaahaaWcbeqaaiabikdaYaaaaeaacqWGRbWAcqGH9aqpcqaIXaqmaeaacqWGobGta0GaeyyeIuoaaSqabaaaaa@46BD@

The slope of the regression line for F(n) on a log scale is calculated (α) and used to estimate the Hurst exponent, hereafter indicated as H_DFA_, with H_DFA _= α-1 for fractional Brownian motion [32].

### Data analysis

Centre of pressure data were calculated from the moment the second foot contacted the force plate (FC_2_) for all displacement directions. The time FC_2 _occurred was calculated as time at which the maximum value of the second derivative of the ML signal occurred, which corresponded to the time the second foot touched the force plate when the largest acceleration of ML would occur when the COP moved rapidly towards the second foot. This instant in time was used for all AP, ML, and R displacements.

Variables were calculated for sliding windows of 10, 5, and 2.5 s, starting from FC_2_. The windows were slid by 1 s increments until nine windows in total were obtained. The number of windows was kept at nine in order to ensure that there were more subjects than windows for subsequent statistical analysis (10 elderly subjects were analysed). The overlap percentage for the three window sizes were 90, 80, and 60% for the windows of 10, 5, and 2.5 s respectively. Estimations of the Hurst exponent were then calculated using DFA and SDA (H_S _and H_L_) methods.

**Figure 1:**
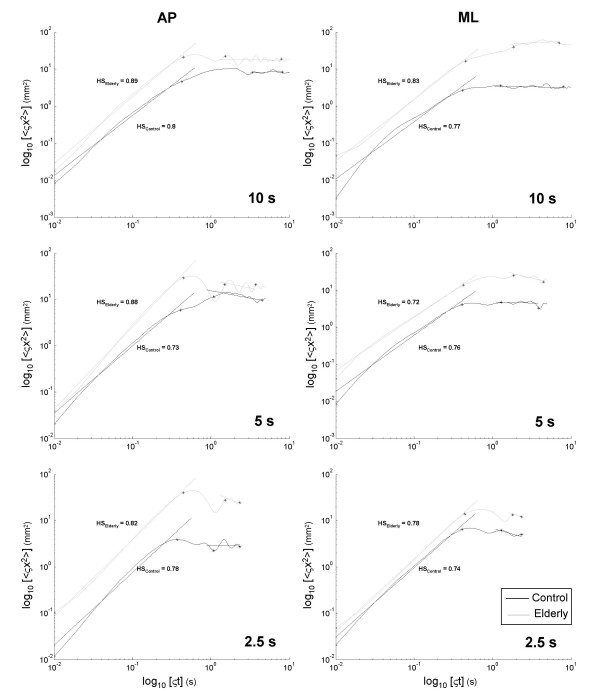
**Stabilogram diffusion analysis plots for elderly and control subjects for anteroposterior and mediolateral displacements**. Data are typical values for an elderly and a control subject for 10, 5, and 2.5 s. Data are plotted in a log-log scale.

**Figure 2:**
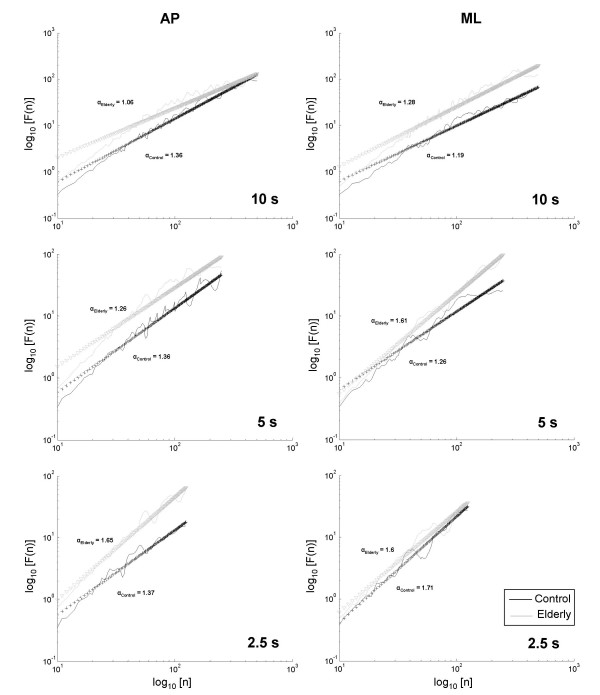
**Detrended fluctuation analysis plots for elderly and control subjects for anteroposterior and mediolateral displacements**. Data are typical values for an elderly and a control subject for 10, 5, and 2.5 s. Data are plotted in a log-log scale.

Examples of SDA and DFA plots calculated for a typical elderly and a typical control subject for all window lengths are presented in Figure 1 and Figure 2, respectively.

All statistical analyses were performed with the Statistical Package for Social Sciences (SPSS Inc., Chicago, IL, USA). Measures of skewness and kurtosis, as well as the Kolmogorov-Smirnov test were used to check for normality [34]. Analysis of variance (ANOVA) was used to test for the effect of subject group on the estimations of the Hurst exponent, with a Bonferroni adjustment when evaluating contrasts. Repeated measures ANOVA was used to test for the effect of the sliding windows on the estimations of the Hurst exponent. The independent variables were subject group and time, with an interaction between subject group and time included. The dependent variables were estimations of the Hurst exponent using the SDA and DFA for the different displacement directions. The intra-class correlation (ICC) was used as a measure of reliability [36], with a two-way mixed model used in order to ensure an unbiased estimation of reliability [37]. Data were expressed as means and 95% confidence intervals. Alpha levels were set at p < 0.05.

## Results

### Sliding window effect

#### Stabilogram Diffusion Analysis (SDA)

There were no differences between the four trials for any of the parameters studied. Accordingly, mean values of all four trials were used for all subsequent statistical analysis, with the notable exception of the reliability analysis.

There were no significant results for H_L _for the effect of time nor were there any differences between window-lengths. In addition values were often less than zero, which would make interpretation difficult. Finally, H_L _was unable to differentiate between subject groups, therefore no further analyses were performed on H_L _and all subsequent references to SDA relate to H_S_.

**Figure 3:**
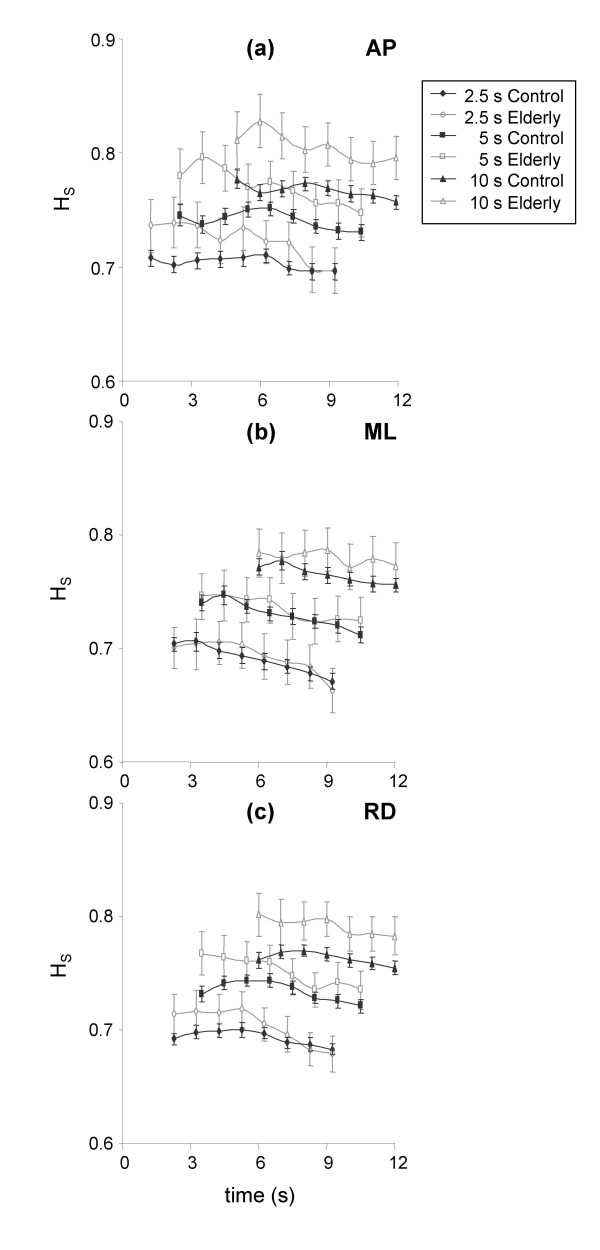
**Evolution of H_S _for anteroposterior (a), mediolateral (b), and resultant (c) displacement**. Data are means and 95% confidence intervals. The x axes represent time in seconds, while the y axes represent the estimation of H_S_. The zero values on the x axes correspond to FC2, while the x coordinate of each data point corresponds to the centre of the data window.

In terms of the effect of time on H_S_, there was a significant decrease for all window lengths for the control group for all displacement directions (Figure 3). In contrast, the effect of time on window length for the elderly group was significant only for the 2.5s window for AP and RD displacement (Figures 3a and 3c). For both the 5 s and 10 s window lengths, although H_S _tended to decrease, the effect was not significant. There were no interaction effects between time and subject group for H_S _for any window length.

#### Detrended Fluctuation Analysis (DFA)

**Figure 4:**
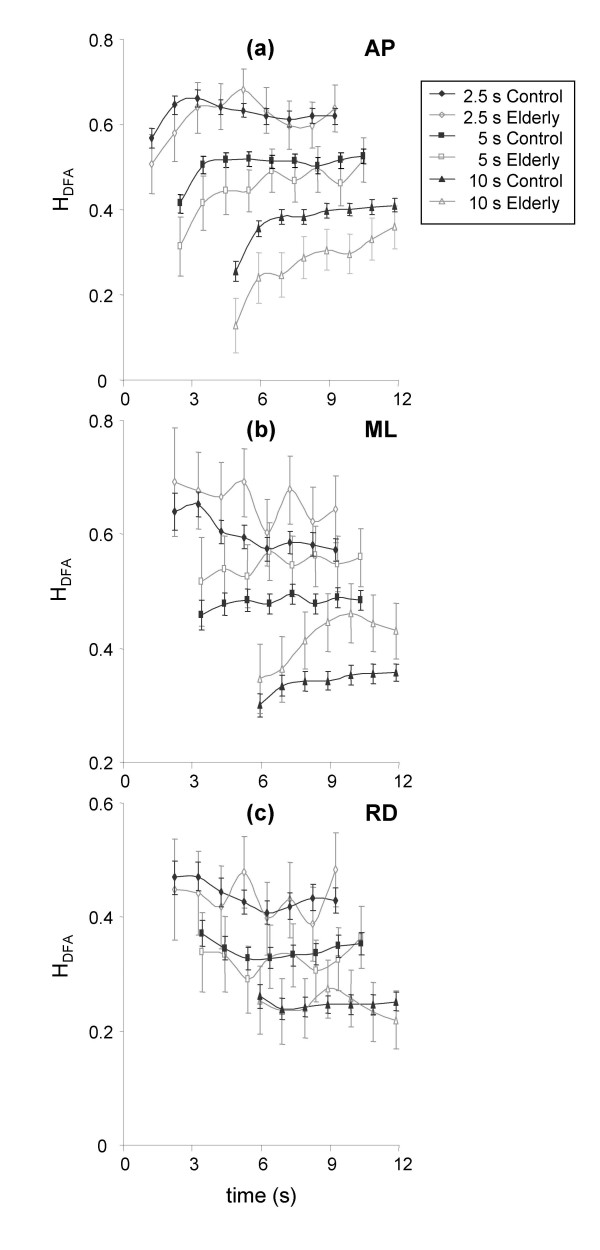
**Evolution of H_DFA _for anteroposterior (a), mediolateral (b), and resultant (c) displacement**. Data are means and 95% confidence intervals. The x axes represent time in seconds, while the y axes represent the estimation of H_DFA_. The zero values on the x axes correspond to FC2, while the x coordinate of each data point corresponds to the centre of the data window.

In terms of the effect of time on H_DFA_, there was a significant increase for all window lengths for the control group for AP displacement (Figure 4a). For ML displacement, H_DFA _decreased for the 2.5-s window, increased for the 10-s window, but did not change significantly for the 5-s window (Figure 4b). For the resultant displacement, H_DFA _decreased significantly for both the 2.5s and 5s window lengths, but not for the 10s window (Figure 4c). For the elderly subjects H_DFA _increased significantly for AP displacement for all window lengths (Figure 4a). In contrast, H_DFA _increased significantly for only the 10s window for ML displacement (Figure 4b), and had no significant change for resultant displacement (Figure 4c).

There was also an interaction effect for all displacement directions for the 10s window, where the rate of increase in H_DFA _was greater for elderly subjects than for the controls.

### Reliability analysis

The reliability analyses were performed separately for the control and elderly subject groups owing to the differences in the values of H_S _and H_DFA _between groups, which are reported in the next section of the results.

#### Stabilogram Diffusion Analysis (SDA)

There was no significant effect of time on the ICC values for any window length. As subsequent tests found no evidence of non-normality, ANOVA was performed on the individual ICC values for each sliding position for each window length in order to identify differences between groups and methods. In respect to differences between the control and elderly subjects, ICC values were significantly higher for the elderly for the 2.5-s window for AP and RD directions, for the 5-s window for all directions, and for the 10-s window for the RD direction (Table 2). In terms of differences between window lengths, the only significant difference was observed for control subjects for the AP direction, whereby the ICC value for the 10-s window was significantly greater than that of the 5-s window (Table 2).

**Table 2:** Mean ICC values for Stabilogram Diffusion Analysis.

	Anteroposterior		Mediolateral		Resultant	
Window size (s)	Control	Elderly	Control	Elderly	Control	Elderly

2.5	0.18	0.55*	0.31	0.34	0.30	0.56*
5	0.29	0.54*	0.42	0.58*	0.43	0.62*
10	0.44^†^	0.49	0.49	0.58	0.53	0.67*

Values are means calculated for all windows of each size.

*Significantly different from control subjects.

†Significantly different from 5-s window.

#### Detrended Fluctuation Analysis (DFA)

As for the SDA, there was no significant effect of time on the ICC values for any window length. Statistical tests were therefore performed on the individual ICC values. Significant differences between the control and elderly subjects were observed for all window sizes for both AP and RD directions (Table 3). In respect to the effect of the window length, the only significant differences were that the ICC values for the 2.5-s window were lower than those for the 5-s window for both AP and ML directions (Table 3).

**Table 3:** Mean ICC values for Detrended Fluctuation Analysis.

	Anteroposterior		Mediolateral		Resultant	
Window size (s)	Control	Elderly	Control	Elderly	Control	Elderly

2.5	0.20^†^	0.56*^†^	0.27^†^	0.34	0.24	0.54*
5	0.40^§^	0.72*^§^	0.41	0.52	0.32^§^	0.56*
10	0.48	0.75*^§^	0.52	0.62	0.43	0.68*

Values are means calculated for all windows of each size.

*Significantly different from control subjects.

†Significantly different from 5-s window.

§Significantly different from the SDA values reported in Table 2.

When ICC values were compared between the SDA and DFA methods, significantly higher values were observed for DFA for both control and elderly subjects for the 5-s window for the AP direction, and for elderly subjects for the AP direction for the 10-s window (Table 2 and 3). In contrast, a significantly higher ICC value was observed for SDA for the RD direction for the 5-s window for control subjects (Table 2 and 3).

### The effect of age on postural stability

#### Stabilogram Diffusion Analysis (SDA)

**Figure 5:**
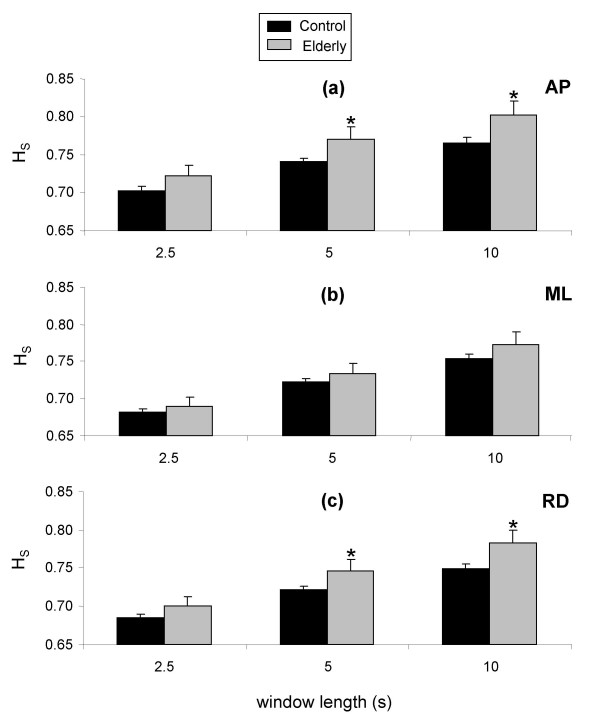
**Differences in H_S _between control and elderly subjects for 2.5-s, 5-s, and 10-s window lengths anteroposterior (a), mediolateral (b), and resultant (c) displacement**. Data are means and 95% confidence intervals for all windows of each window length. *Significantly different from control subjects.

For the analysis of the effect of age on postural stability, mean values across all four tests and all window positions after were obtained for each subject for each window length. These mean values were then used in the subsequent analysis, the results of which are presented in figure 5. It can be seen that no significant differences were observed between groups for ML displacement, regardless of the window size (Figure 5b). However, H_S _calculated for both AP and RD displacement was significantly greater for elderly subjects for both 5 and 10-s windows (Figure 5a and 5c).

#### Detrended Fluctuation Analysis (DFA)

**Figure 6:**
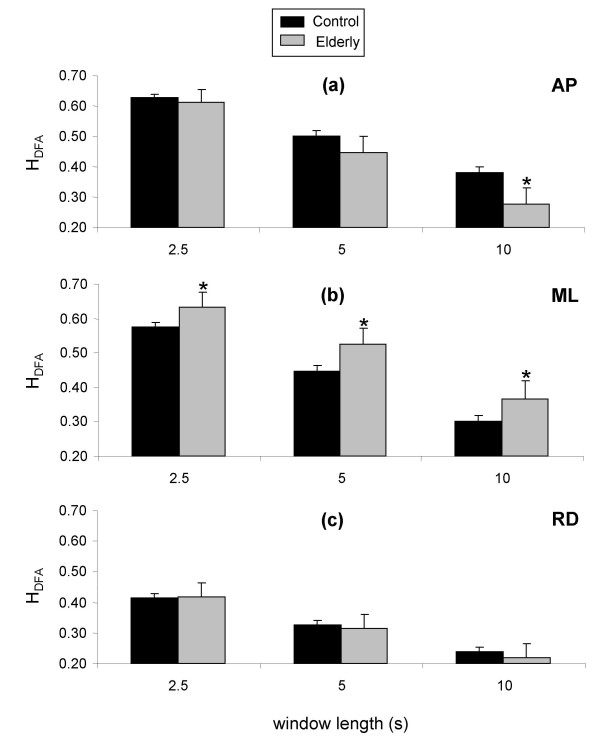
**Differences in H_DFA _between control and elderly subjects for 2.5-s, 5-s, and 10-s window lengths for anteroposterior (a), mediolateral (b), and resultant (c) displacement**. Data are means and 95% confidence intervals for all windows of each window length. *Significantly different from control subjects.

Once again, mean values were obtained across all four tests and all window positions were obtained for each subject for each window length. These mean values were then used in the subsequent analysis, with the results are presented in figure 6. In contrast to the results for SDA presented above, significant differences were observed between groups for ML displacement for all three window sizes, with significantly greater values of H_DFA _observed for elderly subjects (Figure 6b). In respect to AP and RD displacement, the only significant effect of age group on H_DFA _was a decrease in elderly subjects for AP displacement for the 10-s window length (Figure 6a and 6c).

## Discussion

### Sliding window effect

The sliding window analysis was performed in order to identify the optimal time to start analysis. As shown in figure 3, H_S _decreased with time for all displacement directions for control subjects. A decrease in H_S _is indicative of a more precisely controlled movement in that the value of H_S _corresponds to the slope of the short-term of the log-log plot of Δt and <Δx^2^>. This decrease in H_S _is indicative of a more tightly controlled static posture where displacements are smaller for a given time interval. Thus subjects became more stable the longer they remained on the force plate. In contrast, H_S _for elderly subjects decreased only for AP and RD displacement direction, uniquely for the 2.5-s window. The absence of any effect for ML displacement for elderly subjects was due to the increased variability in H_S _for these subjects in comparison to control subjects. Such an explanation is also true for the other displacement directions.

The DFA method yielded similar results to those of the SDA method in that time has a significant effect on H_DFA_. However, in contrast to the results for H_S_, H_DFA _increased with time. In addition, these results were evident for both control and elderly subjects. These results were, however, due to the initial value at FC2, which was much lower than the subsequent values. Given this result it would be worth starting any analysis from FC2 + 1 s in order to remove any effect of time on H_DFA_. The interpretation of H_DFA _depends on the values detected. Values of H_DFA _greater than 0.5 are indicative of a persistent times series, with higher values due to a smoother time series, with a corresponding decrease in variability [38]. As values of H tend towards 1, the signal is smoother with a higher correlation between successive points [39]. High values of H_DFA _would, therefore, be indicative of increased postural stability. Another interpretation is possible for H_DFA _for lower values, whereby H_DFA _less than 0.5 is indicative of an anti-persistent signal. For such data the variation between successive points in the time series is more likely to change direction than to continue in the same direction, thus reflecting a more tightly controlled time series.

#### Reliability analysis

In terms of reliability, the values reported varied according to the window size, the displacement direction, and the subject group, but did not vary with time. In other words, the values of H_S _and H_DFA _for the initial part of the signal were just as reliable as those for the later part where greater postural stability was observed. In respect to the effect of window size, higher ICC values were observed for the 5-s and 10-s window lengths for both SDA and DFA methods. Such a finding was expected given that previous studies of diverse physiological and behavioral time series have typically shown greater variability when less data points are used. Eke and colleagues recommended using time series with at least 2^12 ^(4096) data points due to the unreliable results obtained with shorter time series [32]. However, recent results have demonstrated that, despite an increased variability, time series as short as 2^8 ^(256) points still produced acceptable results [40]. Furthermore, the mean of four short time series of 256 points was shown to provide a better estimate of H than a single time series of 1024 points. The results of the reliability analysis of the present study confirm the results of Delignières, in that ICC values as high as 0.72 were obtained for 5-s time series containing only 500 data points.

Given the low ICC values obtained for the 2.5-s window length, all subsequent comparisons of reliability are discussed for only the 5 and 10-s windows. In respect to differences in reliability for the two groups, it can be seen that elderly subjects were far more reliable than were the control subjects. The ICC values for elderly subjects were consistently considered to be "fair to good", bordering on "excellent" using the scale developed by Fleiss [41], with values varying from 0.49 to 0.75. In contrast, the ICC values for the control subjects were consistently lower than those for the elderly group, ranging from 0.29 to 0.53. Such differences could well be related to the calculation of the ICC:

ICC=(between subject variation−within subject variation)between subject variation
 MathType@MTEF@5@5@+=feaafiart1ev1aaatCvAUfKttLearuWrP9MDH5MBPbIqV92AaeXatLxBI9gBaebbnrfifHhDYfgasaacH8akY=wiFfYdH8Gipec8Eeeu0xXdbba9frFj0=OqFfea0dXdd9vqai=hGuQ8kuc9pgc9s8qqaq=dirpe0xb9q8qiLsFr0=vr0=vr0dc8meaabaqaciaacaGaaeqabaqabeGadaaakeaacqqGjbqscqqGdbWqcqqGdbWqcqGH9aqpdaWcaaqaaiabcIcaOiabbkgaIjabbwgaLjabbsha0jabbEha3jabbwgaLjabbwgaLjabb6gaUjabbccaGiabbohaZjabbwha1jabbkgaIjabbQgaQjabbwgaLjabbogaJjabbsha0jabbccaGiabbAha2jabbggaHjabbkhaYjabbMgaPjabbggaHjabbsha0jabbMgaPjabb+gaVjabb6gaUjabgkHiTiabbEha3jabbMgaPjabbsha0jabbIgaOjabbMgaPjabb6gaUjabbccaGiabbohaZjabbwha1jabbkgaIjabbQgaQjabbwgaLjabbogaJjabbsha0jabbccaGiabbAha2jabbggaHjabbkhaYjabbMgaPjabbggaHjabbsha0jabbMgaPjabb+gaVjabb6gaUjabcMcaPaqaaiabbkgaIjabbwgaLjabbsha0jabbEha3jabbwgaLjabbwgaLjabb6gaUjabbccaGiabbohaZjabbwha1jabbkgaIjabbQgaQjabbwgaLjabbogaJjabbsha0jabbccaGiabbAha2jabbggaHjabbkhaYjabbMgaPjabbggaHjabbsha0jabbMgaPjabb+gaVjabb6gaUbaaaaa@9556@

For heterogeneous subject groups, the between subject variation would increase, thus increasing the ICC, as seen for the elderly subject group. A homogenous subject group would be expected to have less between subject variation, and a resulting decrease in the ICC, as observed for the control group.

In respect to the differences between the SDA and DFA methods in terms of reliability, the DFA method generally produced more reliable parameters for AP displacement, with both ICC values that approached the 0.75 "excellent" level of Fleiss [41] obtained for DFA for elderly subjects for AP displacement. It should be stressed that ICC values calculated for DFA and SDA analysis are only for very short time series. It is likely that higher ICC values would be obtained should longer time series be compared.

When the reliability results are compared with other studies, it is clear that clinical balance tests provide greater reliability, with values as high as 0.99 [42]. However, as the aim of the study is to develop a home-based assessment that does not require the intervention of a third party, a more realistic comparison is that made with other biomechanical measures of balance. In this respect, the ICC values observed are particularly encouraging, especially for elderly subjects, when it is considered that no constraints were imposed on the subjects in terms of foot position. In one study that compared the reliability of SDA parameters, ICC values ranged from 0.41 to 0.79 [43]. The lack of constraint used in the present study was needed in order to ensure that the results could be generalised to a home-based study where it would be impossible to closely control the experimental protocol.

#### The effect of age on postural stability

It was expected that there would be underlying differences between the two subject groups in terms of postural stability. The results of the present study confirmed this assumption for both SDA and DFA methods. Although the two methods both detected differences between the age groups, these differences were not the same for the two methods. The SDA method identified differences in AP displacement provided the window was at least 5-s long. Elderly subjects had increased values of H_S_, which are indicative of a less precisely controlled movement, as outlined at the beginning of the discussion. In contrast, no significant differences were observed for mediolateral displacement using SDA. Elderly subjects also had increased values of H_S _for resultant displacement for the 5-s and 10-s windows. These differences were no doubt due to the differences observed for AP displacement, which is the greatest component of resultant displacement due to the nature of the ankle and knee joints, which limit movement in the mediolateral direction.

In respect to the differences between elderly and control subjects identified by the DFA method, H_DFA _for mediolateral displacement was significantly higher for elderly subjects than for the controls irrespective of window length. As detailed at the start of the discussion section, values of H_DFA _less than 0.5 are indicative of anti-persistence, with lower values reflecting greater anti-persistence, and thus a more closely posture. Elderly subjects were, therefore, less stable than the control subjects for mediolateral displacement. In contrast, H_DFA _values for anteroposterior displacement were lower for elderly subjects than for control subjects, which is indicative of an increased postural stability for elderly subjects in the AP direction.

One interpretation of the two results could be that elderly subjects control their movement in the AP direction more precisely. A similar finding was reported by Norris and colleagues for AP displacement, who identified lower DFA values for elderly subjects at risk of falling [44]. Their interpretation centred around the fact that H_DFA _values were less than 0.5, and thus anti-persistent. The strategy adopted by the elderly subjects was highly anti-persistent, with the aim of reducing AP movement in order to maintain a stable posture.

In contrast to the results of the present study, Norris and colleagues reported no differences in ML displacement between control and elderly subjects. The contrasting findings of the two studies could be due to the different protocols used. In the present study subjects were free to choose their own foot position, values were calculated for analysis windows of 2.5, 5, and 10 s, and analysis commenced as soon as subjects had their two feet on the platform. In contrast Norris and colleagues imposed a standardised foot position, collected data for a 30-s time period, and waited for five seconds after subjects were positioned before beginning data collection. The lack of differences observed may therefore have been due to the imposed condition of a stable posture.

In respect to the differences observed between the SDA and DFA methods, the contrasting findings are due to the method used to analyse the time series. The SDA method provides two estimations of the Hurst exponent, for the short-term (H_S_) and long-term (H_L_) regions of the log-log plot of Δt and <Δx^2^>. Given that it was not possible to exploit the results for H_L_, the SDA method via H_S _provided information that was only related to short-term oscillations. These results indicated persistence, as all values for H_S _were greater than 0.5. In contrast, H_DFA_, which was obtained for the whole time series was less than 0.5, thus demonstrating anti-persistence. Thus, these two methods provide complimentary information related to different aspects of postural control for short-term and long-term auto-correlations.

#### Recording duration

In respect to the minimum recording duration needed, a 5-s window appears sufficient. The 2.5-s window was not sufficiently reliable, whereas reliability was similar for the 5-s and 10-s windows. Differences between subject groups were also evident for both the 5-s and 10-s windows. If the aim is to select the most non-invasive protocol, a 5-s window would be sufficient. It should be noted, however, that decreasing the window length introduces a bias into both H_S _and H_DFA _estimations whereby H_S _is underestimated and H_DFA _is overestimated for short window lengths. Such a finding means that comparison between different populations and different studies would not be possible for different window lengths. However, in terms of a longitudinal home-based study, although a bias would be present for 5-s recordings, the underestimation of H_S _and the overestimation of H_DFA _for short window lengths would not pose a problem for comparison of values between different testing sessions for the same individual given that the measures are reliable. The ideal start point for the analysis would one second after stepping onto the force plate, in order to remove the initial values that were markedly different from subsequent values due to the perturbation induced by the step.

## Conclusion

The SDA and DFA methods were both able to identify differences in postural stability between control and elderly subjects for time series as short as 5 s. In addition, measurements proved to be reliable across testing sessions, with DFA the more robust method for AP displacement. Both methods, as well as providing evidence of underlying postural control strategies, appear to be well-suited to a non-invasive longitudinal assessment of balance.

## Competing interests

The author(s) declare that they have no competing interests.

## Authors' contributions

HA and MA carried out the data collection and data analysis. DH participated in the conception, design, and coordination of the study, performed the statistical analysis, and drafted the manuscript. VM participated in the design and coordination of the study. MD participated in the design and coordination of the study. JD participated in the conception of the study, and its design and coordination. All authors read and approved the final manuscript.
